# Investigating the salmon bias effect among international immigrants in Sweden: a register-based open cohort study

**DOI:** 10.1093/eurpub/ckab222

**Published:** 2022-01-18

**Authors:** Andrea Dunlavy, Agneta Cederström, Srinivasa Vittal Katikireddi, Mikael Rostila, Sol P Juárez

**Affiliations:** 1 Department of Public Health Sciences, Stockholm University and Centre for Health Equity Studies (CHESS), Stockholm University/Karolinska Institutet, Stockholm, Sweden; 2 Institute of Health & Wellbeing, MRC/CSO Social and Public Health Sciences Unit, University of Glasgow, Glasgow, Scotland

## Abstract

**Background:**

Studies of migration and health have hypothesized that immigrants may emigrate when they develop poor health (salmon bias effect), which may partially explain the mortality advantage observed among immigrants in high-income countries. We evaluated the salmon bias effect by comparing the health of immigrants in Sweden who emigrated with those who remained, while also exploring potential variation by macro-economic conditions, duration of residence and region of origin.

**Methods:**

A longitudinal, open cohort study design was used to assess risk of emigration between 1992 and 2016 among all adult (18+ years) foreign-born persons who immigrated to Sweden between 1965 and 2012 (*n* = 1 765 459). The Charlson Comorbidity Index was used to measure health status, using information on hospitalizations from the Swedish National Patient Register. Poisson regression models were used to estimate incidence rate ratios (RRs) with 95% confidence intervals (CIs) for emigrating from Sweden.

**Results:**

Immigrants with low (RR = 0.83; 95% CI: 0.76–0.90) moderate (RR = 0.70; 95% CI: 0.62–0.80) and high (RR = 0.62; 95% CI: 0.48–0.82) levels of comorbidities had decreased risk of emigration relative to those with no comorbidities. There was no evidence of variation by health status in emigration during periods of economic recession or by duration of residence. Individuals with low to moderate levels of comorbidities from some regions of origin had an increased risk of emigration relative to those with no comorbidities.

**Conclusions:**

The study results do not support the existence of a salmon bias effect as a universal phenomenon among international immigrants in Sweden.

## Introduction

Migration is a dynamic process that is influenced by a confluence of factors related to the country of origin, the migration experience and the settlement context; yet, the health of immigrants is also intertwined with migration, as it influences both ability and decisions to migrate. Previous research has shown that international immigrants have a mortality advantage relative to native-born majority populations in high-income countries,^1^ commonly referred to as the *healthy migrant hypothesis*^2^ or *healthy migrant paradox.*^3^ Different explanations have been put forth for this relative advantage, including positive health selection of immigrants in the country of origin, as well as negative health selection in emigration, whereby it has been hypothesized that as immigrants develop poor health, they may be more likely to emigrate (*salmon bias effect)*,^4^ in order to receive care or support from family in more familiar or comfortable environments^5^^,^^6^ or health care services,^7^ leading to a positive health selection among those who remain.

However, empirical evidence supporting the salmon bias effect is limited,^8–10^ especially in settlement contexts outside of the USA, where the salmon bias effect has been the most extensively studied among Hispanic immigrants, but for which evidence supporting this effect has also been mixed.^7^^,^^11–13^ Many previous studies have indirectly tested the salmon bias effect by comparing mortality rates among groups of international immigrants with differing feasibility or probability of emigration.^14^^,^^15^ Others have focussed on internal migrants in order to overcome censoring bias related to unrecorded e-migration,^9^^,^^16^^,^^17^ whereby individuals who have actually emigrated continue to be registered as residents and included in administrative registers, which can lead to an underestimation of mortality estimates as a result of numerator–denominator mismatch.^18^^,^^19^ Only a paucity of empirical research has evaluated the health status of international immigrants who emigrate. A Danish study^20^ showed a lower risk of emigration among refugee and family reunification immigrants with greater disease severity relative to immigrants without disease. However, the generalization of these findings to other immigrant groups is unclear. For instance, immigrants coming from countries in conflict may be less likely to emigrate than labour migrants, thereby also being less likely to contribute to denominator bias, but may have fewer guarantees of receiving medical assistance in their home countries.^21^ The likelihood of emigration may also be influenced by macro-economic conditions (e.g. recessionary periods) in the settlement context, as immigrants, especially newcomers, are more likely to face disadvantages in the labour market compared with native-born majority populations.^22^ Depending on the feasibility of international mobility as well as education and professional and language skills, some immigrants may have greater employment opportunities elsewhere; as such, recent immigrants and immigrants with free movement rights [e.g. European Union (EU) citizens] may be more likely to emigrate than more established immigrants.

In this study, we aim to evaluate the salmon bias effect among international immigrants in Sweden by assessing if there are systematic differences in health among immigrants who have emigrated compared with those who remain in the country. Effect modification by macro-economic periods of recession, duration of residence, and region of origin will be considered, as these factors may influence associations between health status and emigration.

## Methods

### Study population and study design

Administrative data from multiple population-based registers which were linked via pseudonymized personal identification numbers were used to define the study population. An open cohort study design was utilized to allow for the inclusion of individuals who immigrated to Sweden during the study period. All foreign-born persons who immigrated to Sweden between 1965 and 2012 and were 18+ years old during the follow-up period (1992–2016) were included.

The study population was additionally categorized into nine regions of origin groups, using predefined country and region of origin categories constructed by Statistics Sweden (see table 1). Persons from Finland comprised a large proportion of the study population and were analyzed as a separate group. Additional groups included: *other Nordic countries* (excluding Finland), *other EU-28 countries* (excluding all Nordic countries but including the UK and Northern Ireland, who were members of the EU during the study follow-up), *Eastern Europe and Russia* (including former Yugoslavia), *North America and Oceania*, the *Middle East* (including Turkey), *Asia*, *Horn of Africa*, *Rest of Africa* and *South America*. Oceania was comprised largely of individuals from Australia, and as such was grouped with North America due to the similar economic development contexts of these two groups.

**Table 1:** Descriptive characteristics of the study population

	***n*** (%)	Person-time/ 1000 py	Recorded emigrations	Unrecorded emigrations	Total emigrations	Total emigration rates/1000 py
Sex						
Men	896 232 (50.8)	9960.5	210 165	11 591	221 756	22.3
Women	869 227 (49.2)	10 692.6	168 918	6168	175 086	16.4
Region of origin						
Finland	166 286 (9.4)	2855.6	39 255	566	39 821	13.9
Other Nordic countries	138 243 (7.8)	1386.2	64 526	2044	66 570	48.0
Other EU28 countries	169 856 (9.6)	1642.1	58 917	2626	61 543	37.5
Eastern Europe and Russia	347 270 (19.7)	4876.8	44 729	3655	48 384	9.9
North America and Oceania	61 072 (3.5)	566.4	26 501	762	27 263	48.1
Middle East	377 689 (21.4)	4286.2	42 070	2672	44 742	10.4
Asia	254 750 (14.4)	2352.1	57 838	2921	60 759	25.8
Horn of Africa	93 281 (5.3)	808.0	12 216	938	13 154	16.3
Rest of Africa	78 300 (4.4)	775.2	16 119	875	16 994	21.9
South America	78 712 (4.5)	1104.5	16 912	700	17 612	15.9
Age						
18–25		2176.3	48 326	3064	51 390	23.6
26–35		4761.5	149 279	5923	155 202	32.6
36–45		4864.6	96 321	3635	99 956	20.5
46–55		3998.9	44 354	2791	47 145	11.8
56–65		2610.5	21 708	1263	22 971	8.8
66–75		1426.0	12 775	720	13 495	9.5
76+		815.3	6355	363	6718	8.2
Period						
1992–96		2925.4	55 216	709	55 925	19.1
1997–2002		4140.9	68 739	1628	70 367	17.0
2003–07		4093.4	76 329	3453	79 782	19.5
2008–11		3888.1	71 724	7278	79 002	20.3
2012–16		5605.3	107 110	4691	111 801	19.9
Duration of residence						
0–5 years		5541.1	232 801	12 053	244 854	44.2
6–10 years		2877.4	56 830	2072	58 902	20.5
11–15 years		2529.3	31 346	1209	32 555	12.9
16–20 years		2486.2	22 907	963	23 870	9.6
20+ years		7219.0	35 234	1462	36 696	5.1

py, person-years.

Additional covariates, including sex (men and women), age (18–25; 26–35; 36–45; 46–55; 56–65; 66–74; and 75+ years), and duration of residence (under 5 years; 5–10; 10–15; 15–20; and more than 20 years) were also assessed. The follow-up period was sub-divided into five period factors (1992–96; 1997–2002; 2003–07; 2008–11; and 2012–16) to account for macro-economic conditions in Sweden, where periods of economic recession (1992–96; 2008–11), recovery (1997–2002; 2012–16) and stability (2003–07) were considered.

### Emigration

The outcome was defined as the first emigration from Sweden in the follow-up period 1992–2016. Emigration from Sweden was defined in two ways: (i) as the first recorded date of emigration from the Register of the Total Population^23^ and (ii) proxy measured using information on sources of income (job-earnings or social benefits) from the Longitudinal Integration Database for Health and Labour Market Studies register,^24^ whereby persons who did not have registered sources of income for 2 consecutive years (within each sub-divided follow-up period) were considered as having emigrated at the midpoint of the period factor. Previous studies from the Swedish context have used similar register-based information on income^18^^,^^25–27^ to account for unrecorded emigration, as a consistent lack of income indicates that an individual is not resident in the country. Although *a priori* health differences between immigrants with recorded and unrecorded emigration are not expected, utilization of the proxy measure allows for an assessment of unrecorded emigration, which will decrease the likelihood of denominator bias. Individuals who emigrated before the start of follow-up were excluded.

### Health status

The main exposure of interest was the Charlson Comorbidity Index (CCI),^28^ a categorization measure of comorbidities that is calculated using International Classification of Diseases diagnosis codes. Annual information on reason for inpatient care (hospitalizations) from the National Patient Register^29^ during the 2 years before the start of each period factor was used to create comorbidity scores based on the number and type of hospitalizations for multiple categories of disease (see Supplementary table S1). Each disease category was weighted based on severity (1–6), and the sum of all weights was used as the overall score for each individual within each period factor. The CCI scores were categorized and ranged from 0 to 3+. Scores of zero indicated no comorbidity (no hospitalizations), and scores of 3+ indicated the highest disease severity.

### Statistical analyses

Poisson regression models were used to derive incidence rate ratios (RRs) with 95% confidence intervals (CIs), with age, calendar time (period factors), and duration of residence used as the three relevant time-scales. The RR of emigration was assessed using both recorded and unrecorded emigrations. Sensitivity analyses which included only recorded emigrations showed comparable findings, with the exception of emigration during the period 2008–11, during which a slightly lower risk of emigration was observed when only recorded emigrations were considered (see Supplementary figure S2). All models were adjusted for sex, age, period factors, duration of residence and region of origin, with robust standard errors. We formally tested for interactions between the CCI and (i) macro-economic period factors, (ii) duration of residence and (iii) region of origin using likelihood-ratio tests between models with and without multiplicative interaction terms. All analyses were conducted in R version 4.0.3 (R Core Team) with the use of Lexis splits from the Epi package 2.42 (Bendix Carsten, Martyn Plummer).

## Results

Table 1 describes the key study variables. Emigration rates were higher among men [22.3/1000 person-years (py)] than women (16.4/1000 py) and varied by region of origin. The lowest rates of emigration were observed among those from Eastern Europe and Russia (9.9/1000 py) and the Middle East (10.4/1000 py), whereas the highest rates were seen among those from other Nordic countries (48.0/1000 py) and North America and Oceania (48.1/1000 py). Emigration rates were stable across all period factors, ranging from 17.0/1000 py during the period 1997–2002, and 20.3/1000 py during the period 2008–11. Rates of emigration were lowest among immigrants who had resided in Sweden for 20+ years (5.1/1000 py) and highest among those with 5 years or less of residence (44.2/1000 py).

**Figure 1:**
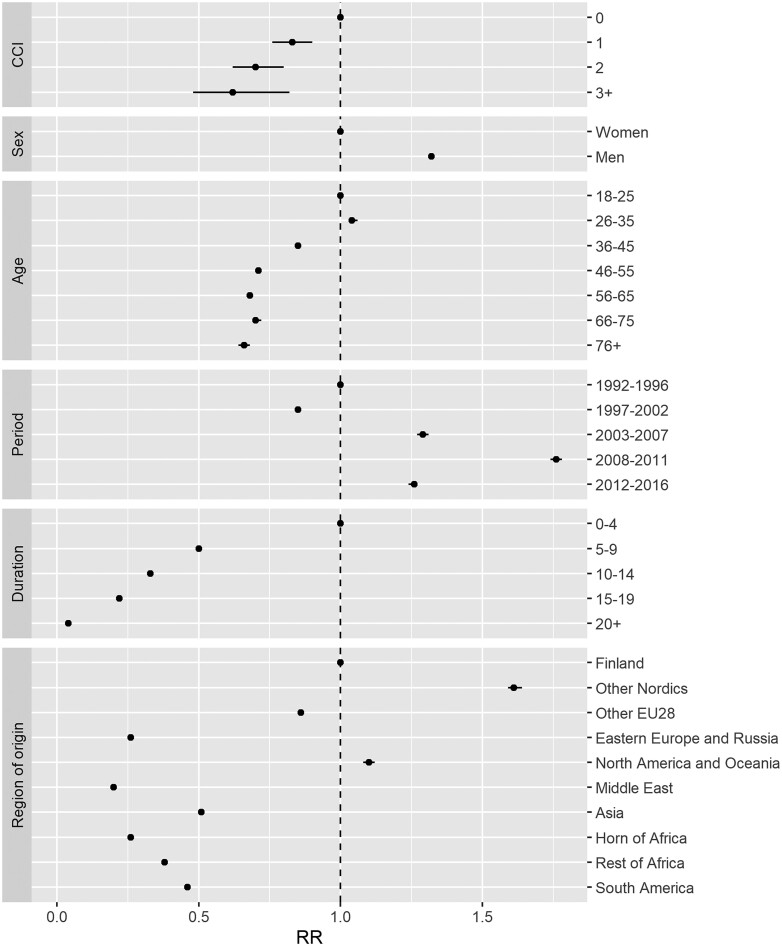
Incidence RRs with 95% CIs for emigration among immigrants to Sweden by CCI score. Adjusted for sex, age, macroeconomic period factors, duration of residence and region of origin

Figure 1 shows the association between emigration and health status as measured by the CCI during the entire follow-up period. A gradient in the relative risk of emigration was observed whereby emigration risk was significantly lower among immigrants who suffered from comorbidities relative to those who did not. Emigration risk was higher among men (RR = 1.32, 95% CI: 1.31–1.33) relative to women, and was lower for immigrants over age 35 relative to the youngest immigrants in the study population. Supplementary table S2 displays the data from figure 1 in tabular format.

Increased risks of emigration were observed during all macro-economic period factors after 2002, with the largest risk of emigration observed during the period 2008–11 (RR = 1.76, 95% CI 1.74–1.78). Although there were period effects in the risk of emigration, with emigration most likely to occur during the years surrounding the 2008 financial crisis, the interaction analysis indicated that the association between health status and emigration was not modified by macroeconomic period (*P* = 0.63).

A gradient in the relative risk of emigration was also seen by duration of residence, whereby those with longer duration of residence (over 5 years) had a lower risk of emigration than those with established residence in Sweden for 5 years or less. The interaction analysis demonstrated that duration of residence did not modify the association between health status and emigration (*P* = 0.56).

**Figure 2:**
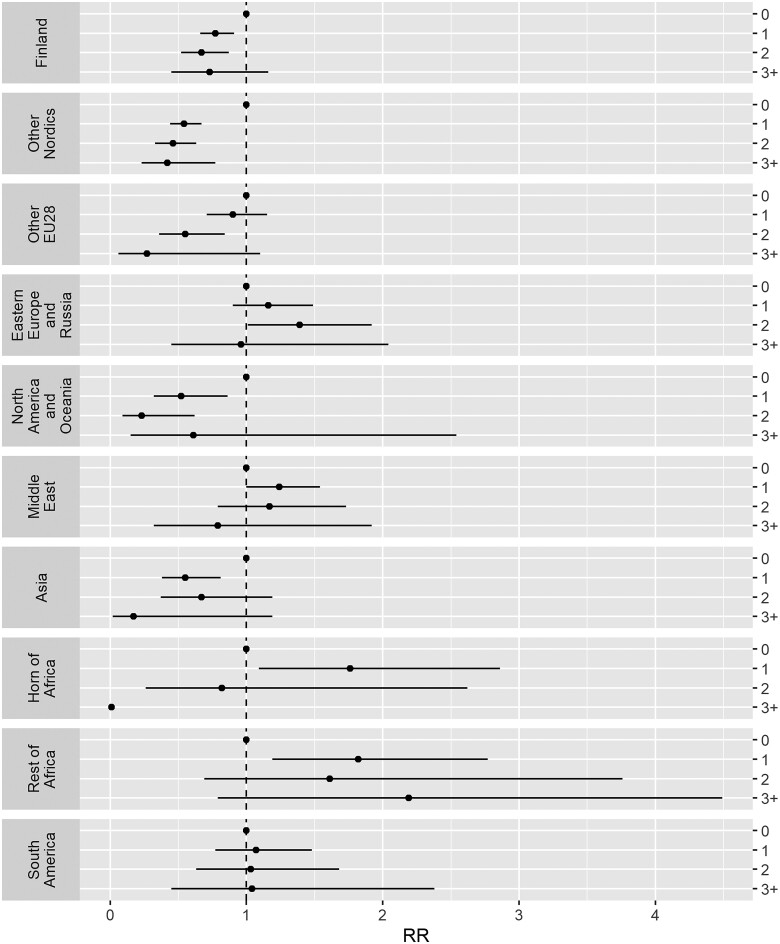
Incidence RRs with 95% CIs for emigration by CCI scores and region of origin. Adjusted for sex, age, macroeconomic period factors and duration of residence

Relative to immigrants from Finland, most region of origin groups showed a lower risk of emigration, except those from other Nordic countries and those from North America and Oceania. The interaction analysis indicated that the association between health status and emigration varied by region of origin (*P* < 0.001). Figure 2 shows the risk of emigration among individuals within each region of origin category, using those with no disease comorbidity (CCI score = 0) from each region of origin as the reference. In most region of origin groups, those with higher disease severity demonstrated an equal or lower risk of emigration than those with no comorbidities. Exceptions to this pattern included immigrants with low disease comorbidity (CCI score = 1) from the Middle East (RR = 1.24; 95% CI: 1.00–1.54), the Horn of Africa (RR = 1.76, 95% CI: 1.09–2.86) and the Rest of Africa (RR = 1.82; 95% CI : 1.19–2.77) as well as immigrants with moderate disease comorbidity (CCI score = 2) from Eastern Europe and Russia (RR = 1.39; 95% CI : 1.01–1.92), all of whom demonstrated an increased risk of emigration relative to those from the same region of origin with no comorbidity. Risk of emigration could not be assessed among immigrants with the highest disease comorbidity (CCI score = 3+) from the Horn of Africa due to insufficient data for analysis among this group. Supplementary table S3 shows the data from figure 2 in tabular form.

## Discussion

This study demonstrated that immigrants in Sweden with low, moderate and high disease comorbidity generally had lower risks of emigration relative to immigrants without comorbidities, which challenges the salmon bias effect. These results were also consistent when considering only recorded emigrations. Furthermore, while periods of economic crisis and length of residence were shown to impact the likelihood of emigration in immigrant populations in Sweden, there was no evidence of effect modification by health status in emigration during times of economic recession or by duration of residence. When interactions between health status and region of origin were investigated, in the majority of region of origin groups individuals with comorbidities showed an equal or lower risk of emigration relative to those without disease comorbidity, although there were variations.

Our overall results were largely in line with those of a Danish study^20^ which found a lower risk of emigration among refugee and family reunification immigrants with poorer health status. Our study also used proxy measures to account for unrecorded emigration to minimize the risk of denominator bias. The comparability of our results which included both recorded and unrecorded emigration relative to those which only included recorded emigrations suggested that such bias was not present in our findings, and that there was no systematic variation in health status when comparing immigrants with recorded vs. unrecorded emigration. Taken together, our findings provide additional evidence to suggest that the salmon bias effect is not a key driver of the mortality health advantage observed among the majority of immigrant groups in Sweden.^27^^,^^30^

However, immigrants from Finland are a group which has been shown to deviate from this pattern of advantage, with several studies rather demonstrating elevated mortality risks^27^^,^^31^^,^^32^ as well as severe morbidities, including increased risk of hospitalizations for alcohol-related disorders^33^ and myocardial infarctions.^34^ As such, the lower risk of emigration found among persons from Finland with comorbidities (i.e. CCI score of 1–3+) relative to those with no comorbidities, could to some extent partially explain the previously observed elevated mortality risks in this group. Furthermore, the overall finding that immigrants in general with more comorbidities were less likely to emigrate than those without comorbidities, irrespective of their duration of residence, suggests that any indication of worse health with increasing duration of residence (a phenomenon commonly referred to as *unhealthy assimilation*)^35^ was likely to be a true health effect, and not an artefact due to unhealthy selective emigration.

Interaction analysis of the risk of emigration by health status and region of origin did not show a uniform pattern of findings. Yet, there was an indication pointing towards an association between the level of emigration rates and health status: the groups of immigrants showing the strongest evidence against the salmon bias effect were generally those with higher emigration rates, the exception being immigrants from Finland, with relatively low emigration rates (14%). Health status did not appear to influence risk of emigration in immigrants from South America. However, there was some evidence of a salmon bias effect among persons from the Horn of Africa, the Rest of Africa, the Middle East and Eastern Europe and Russia, whereby those with low to moderate disease comorbidity showed an elevated, rather than a decreased or similar, risk of emigration relative to those with no disease. These groups also had lower rates of emigration in general. However, this effect was not demonstrated amongst those with the highest disease severity (CCI = 3+), and as such evidence supporting the salmon bias effect remains limited. Furthermore, data on the country of emigration was not available in this study, and it remains unknown if individuals who emigrated returned to their countries of origin or moved to a third country. There is existing evidence showing patterns of emigration from Sweden to other countries in Europe, where access to universal health care is often guaranteed, among immigrants who also have EU citizenship or freedom of movement rights.^36–38^ As such, emigration may be more likely to occur among some individuals with low to moderate ill-health, for whom migration is feasible and where necessary or desired care in the country of emigration can be guaranteed. Other research has postulated that persons who face economic or social adversities in settlement and subsequently might also be in poorer health may likewise emigrate to seek out alternative employment opportunities.^7^ Further research is needed to explore these findings.

### Strengths and limitations

This study utilized population-based register data that enabled us to longitudinally assess the risk of emigration in a large study population of international immigrants in Sweden. We were able to use these data to account for unrecorded emigrations, a key limitation of previous studies. We were also able to account for additional factors that can influence decisions to emigrate, including periods of economic crisis and duration of residence. Despite these strengths, our study was also tempered by some limitations. First, we were not able to specifically assess immigrants’ reason for migration to Sweden, but rather assessed region of origin, which has been widely used as a proxy measure of reason for migration in previous research, but also has several limitations.^39^ However, a previous Swedish study^40^ on risk of adverse birth outcomes among immigrant mothers suggested that administrative information on reason for migration (refugee vs. non-refugee) was not relevant due to its lack of specificity, as family reunification immigrants from countries in conflict may often be categorized as non-refugee immigrants. Relatedly, the generalizability of our findings may be limited to countries which provide universal access to health care services to all legal residents, such as Sweden. Immigrants residing in country contexts that do not provide such access may be more likely to emigrate if they require medical care that could be provided in the country of origin or a third country.

## Conclusions

Decisions to migrate are complex and influenced by a number of social, economic and health-related factors. This study has provided evidence which challenges the salmon bias effect, as our findings did not universally support the existence of unhealthy selective emigration among international immigrants in Sweden. Future research is needed to investigate alternative explanations for the mortality advantage often observed among immigrants, including consideration of both protective and risk factors related to the origin and settlement contexts, and should continue to explore potential drivers behind health selection effects in emigration. 

## Supplementary data

Supplementary data are available at *EURPUB* online.

## Ethics approval

This study was approved by the Regional Ethical Review Board of Stockholm (decision no. 2017/716-31/5).

## Data availability

The register data underlying this article cannot be shared publicly as it contains sensitive personal information. Access to the register data can be applied for by contacting the relevant Swedish public authorities, including Statistics Sweden and the Swedish National Board for Health and Welfare.

## Funding

This work was supported by The Swedish Research Council for Health, Working Life and Welfare (Forte) [grant number 2016-071289]. SPJ acknowledges funding from The Swedish Research Council for Health, Working Life and Welfare (Forte) [grant number 2021-00271] and SPJ and AD from the Swedish Research Council (VR) [grant number 2018-01825]. SVK acknowledges funding from an NRS Senior Clinical Fellowship [SCAF/15/02], the UK Medical Research Council [MC_UU_00022/2] and Scottish Government Chief Scientist Office [SPHSU17].

*Conflict of interest*: None declared.

Key points:

- Immigrants in Sweden with low, moderate and high levels of comorbidities had a lower risk of emigration than immigrants with no comorbidities, as measured by the Charlson Comorbidity Index.
- Results were comparable when only recorded emigrations were considered.
- Periods of economic recession and length of duration of residence did not modify the association between health status and emigration.
- There was some evidence of modification in the association between health status and emigration in some region of origin groups, whereby persons with low to moderate levels of comorbidities showed an increased risk of emigration relative to those from the same region of origin without comorbidities.

## Supplementary Material

ckab222_Supplementary_Data
