# Supplementary material for: Investigating the salmon bias effect among international immigrants in Sweden: a register-based open cohort study
Source: Eur J Public Health. 2022 Jan 18;32(2):226–32. doi: 10.1093/eurpub/ckab222 (PMC8975526; doi:10.1093/eurpub/ckab222)
Supplement: ckab222_Supplementary_Data [file ckab222_supplementary_data.docx]

Supplementary Table S1. ICD-9 and ICD-10 diagnostic codes and weights used for the Charlson Comorbidity Index (CCI) scoring.

| Comorbid condition | ICD-9 codes | ICD-10 codes | Weight |
| --- | --- | --- | --- |
| Cerebrovascular disease | 430-438; 3526; 3623 | G45; G46; H34.0; I6 | 1 |
| Chronic pulmonary disease | 114; 490-496; 500-505; 4168; 4169; 4789; 4930;  4931; 4939; 5060; 5064;  5081; 5088 | I27.8; I27.9; J40-J47: J60-J67; J68.4; J70.1; J70.3 | 1 |
| Congestive heart failure | 402; 3989; 4041; 4049;  4252; 4254; 4255; 4259;  4280; 4281; 4289; 4299;  7798; 4220A; 4257A; 4258A | I09.9; I11.0; I13.0; I13.2; I25.2; I42.0; I42.5-I42.9; I43; I50; P29.0 | 1 |
| Dementia | 290; 2941; 2953; 3101; 3310; 3312 | F00-F03; F05.1; G30; G31.1 | 1 |
| Diabetes without chronic complications | 2500-2502; 2509; 2510; 2518 | E10.0; E10.1; E10.6; E10.8; E10.9; E11.0; E11.1; E11.6; E11.8; E11.9; E12.0; E12.1; E12.6; E12.8; E12.9; E13.0; E13.1; E13.6; E13.8; E13.9; E14.0; E14.1; E14.6; E14.8; E14.9 | 1 |
| Mild liver disease | 571; 702-706; 709; 5728; 5733; 5734; 5738; 5739; 5761; V427 | B18; K70.0-K70.3; K70.9; K71.3-K71.5; K71.7; K73; K74; K76.0; K76.2-K76.4; K76.8; K76.9; Z94.4 | 1 |
| Myocardial infarction | 410; 412 | I21; I22; I25.2 | 1 |
| Peptic ulcer disease | 531; 532; 533; 534; 5378; 5698 | K25-K28 | 1 |
| Peripheral vascular disease | 441; 4400; 4401; 4402; 4408; 4409; 4431; 4438; 4439; 4471; 5570; 5571; 5579; 4438A; V432; V434 | I70; I71; I73.1; I73.8; I73.9; 177.1; 179.0; 179.2; K55.1; K55.8; K55.9; Z95.8; K95.9 | 1 |
| Rheumatic disease | 725; 7100; 7101; 7103; 7104; 7108; 7140; 7141; 7142; 7148; 7149 | M05; M06, M31.5, M32-M34; M35.1; M35.3; M36.0 | 1 |
| Any malignancy | 140-165; 170-175; 179; 180-195; 200-208; 2362; 2386; 2387; 2732; 2733; 2898; 7573 | C0; C1; C20-C26; C30-C34; C37-C41; C43; C45-C58; C6; C70-C76; C81-C85; C88; C90-C97 | 2 |
| Diabetes with chronic complications | 2503-2507 | E10.2-E10.5; E10.7; E11.2-E11.5; E11.7; E12.2-E12.5; E12.7; E13.2-E13.5; E13.7; E14.2-E14.5; E14.7 | 2 |
| Hemiplegia or paraplegia | 344; 3341; 3352; 3420; 3421; 3429; 3431; 3432; 3434 | G04.1; G11.4; G80.2; G81; G82; G83.0-G83.4; G83.9 | 2 |
| Renal disease | 403; 585; 586; 4040; 5820; 5821; 5822; 5828; 5830; 5831; 5832; 5838; 5880; 5939; 7919; V451; V560; V568 | I12.0; I13.1; N03.2-N03.7; N05.2-N05.7; N18; N19; N25.0; Z49.0-Z49.2; Z99.2 | 2 |
| Moderate or severe liver disease | 570; 4560; 4561; 4568;  5722-5724; 4562A | I85.0; I85.9; I86.4; I98.2; K70.4; K71.1; K72.1; K72.9; K76.5-K76.7 | 3 |
| HIV/AIDS | 2796 | B20-B24 | 6 |
| Metastatic solid tumour | 196-199; 2389 | C77-C80 | 6 |

Supplementary Figure S2. Incidence rate ratios with 95% CI for emigration, comparing recorded emigrations only and combined recorded and unrecorded emigrations. **^a^**


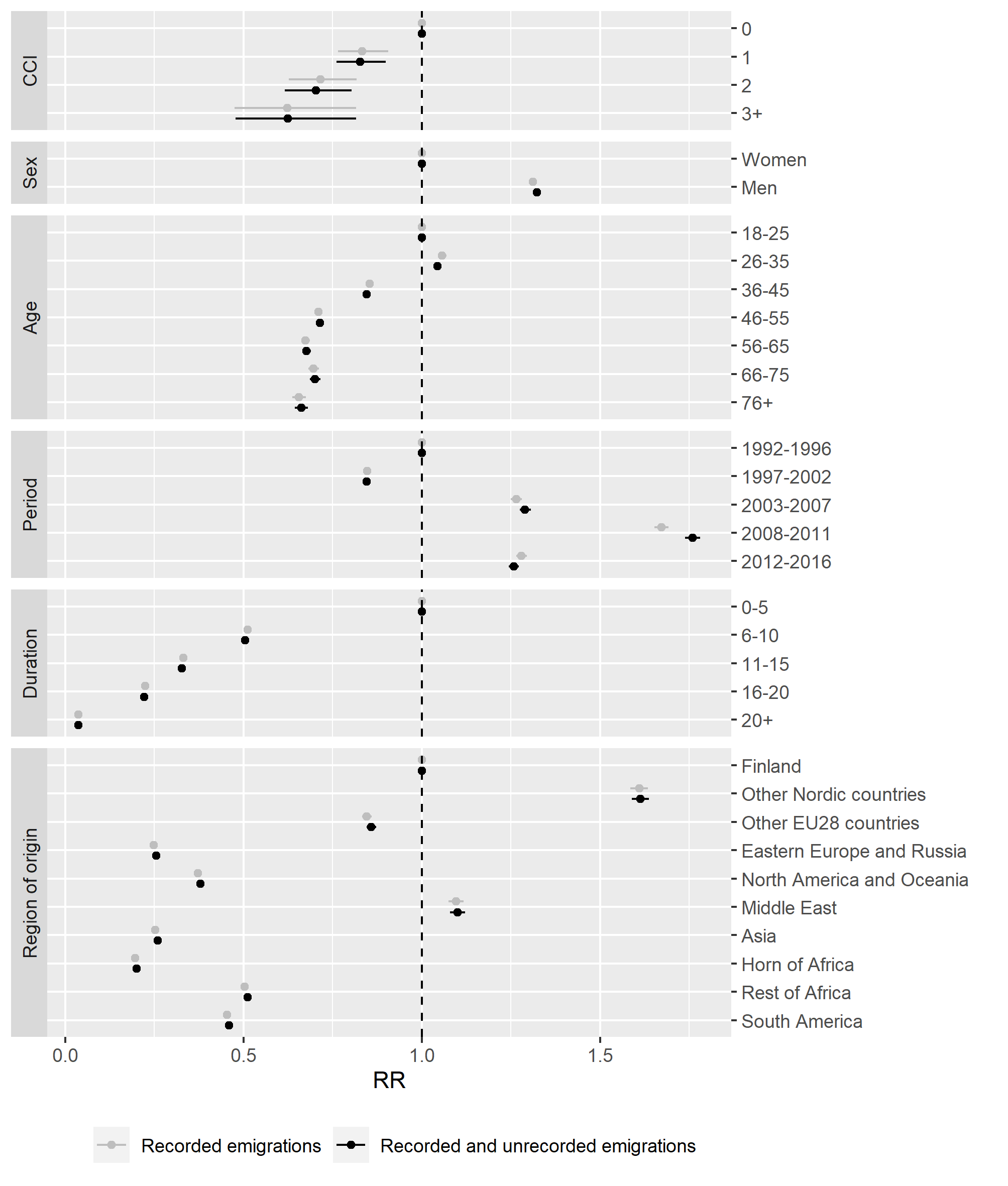


**^a^** adjusted for sex, age, macroeconomic period factors, duration of residence, and region of origin.

^b^ CCI= Charlson Comorbidity Index; Duration= duration of residence

Supplementary Table S3. Incidence rate ratios (RR) with 95% Confidence intervals (CI) for emigration among immigrants by Charlson Comorbidity Index (CCI) score, tabular format of data from Figure 1.

|  | RR | 95% CI | p-value |
| --- | --- | --- | --- |
| **Charlson Comorbidity Index (CCI)** |  |  |  |
| 0 | 1.00 |  |  |
| 1 | 0.83 | 0.76-0.90 | < 0.001 |
| 2 | 0.70 | 0.62-0.80 | < 0.001 |
| 3+ | 0.62 | 0.48-0.82 | < 0.001 |
| **Sex** |  |  |  |
| Women | 1.00 |  |  |
| Men | 1.32 | 1.31-1.33 | < 0.001 |
| **Age (years)** |  |  |  |
| 18-25 | 1.00 |  |  |
| 26-35 | 1.04 | 1.03-1.06 | < 0.001 |
| 36-45 | 0.85 | 0.84-0.86 | < 0.001 |
| 46-55 | 0.71 | 0.70-0.72 | < 0.001 |
| 56-65 | 0.68 | 0.67-0.69 | < 0.001 |
| 66-75 | 0.70 | 0.69-0.72 | < 0.001 |
| 76+ | 0.66 | 0.64-0.68 | < 0.001 |
| **Period factors** |  |  |  |
| 1992-1996 | 1.00 |  |  |
| 1997-2002 | 0.85 | 0.84-0.86 | < 0.001 |
| 2003-2007 | 1.29 | 1.27-1.31 | < 0.001 |
| 2008-2011 | 1.76 | 1.74-1.78 | < 0.001 |
| 2012-2016 | 1.26 | 1.24-1.27 | < 0.001 |
| **Duration of residence (years)** |  |  |  |
| 0-5 | 1.00 |  |  |
| 6-10 | 0.50 | 0.50-0.51 | < 0.001 |
| 11-15 | 0.33 | 0.32-0.33 | < 0.001 |
| 16-20 | 0.22 | 0.22-0.22 | < 0.001 |
| 20+ | 0.04 | 0.04-0.04 | < 0.001 |
| **Region of origin** |  |  |  |
| Finland | 1.00 |  |  |
| Other Nordic countries | 1.61 | 1.59-1.64 | < 0.001 |
| Other EU28 countries | 0.86 | 0.85-0.87 | < 0.001 |
| Eastern Europe and Russia | 0.26 | 0.25-0.26 | < 0.001 |
| North America and Oceania | 1.10 | 1.08-1.12 | < 0.001 |
| Middle East | 0.20 | 0.20-0.20 | < 0.001 |
| Asia | 0.51 | 0.50-0.52 | < 0.001 |
| Horn of Africa | 0.26 | 0.25-0.27 | < 0.001 |
| Rest of Africa | 0.38 | 0.37-0.39 | < 0.001 |
| South America | 0.46 | 0.45-0.47 | < 0.001 |

**^a^** adjusted for sex, age, macroeconomic period factors, duration of residence, and region of origin.

Supplementary Table S4. Incidence rate ratios (RR) with 95% Confidence Intervals (CI) for emigration by Charlson Comorbidity Index (CCI) scores and region of origin, **^a^** tabular format of data from Figure 2.

|  | **RR** | **95% CI** | **p-value** |
| --- | --- | --- | --- |
| **Finland** |  |  |  |
| CCI score=0 | 1.00 |  |  |
| CCI score=1 | 0.77 | 0.66-0.91 | 0.002 |
| CCI score=2 | 0.67 | 0.52-0.87 | 0.002 |
| CCI score=3+ | 0.73 | 0.45-1.16 | 0.183 |
| **Other Nordic countries** |  |  |  |
| CCI score=0 | 1.00 |  |  |
| CCI score=1 | 0.54 | 0.44-0.67 | < 0.001 |
| CCI score=2 | 0.46 | 0.33-0.63 | < 0.001 |
| CCI score=3+ | 0.42 | 0.23-0.77 | 0.005 |
| **Other EU 28 countries** |  |  |  |
| CCI score=0 | 1.00 |  |  |
| CCI score=1 | 0.90 | 0.71-1.15 | 0.416 |
| CCI score=2 | 0.55 | 0.36-0.84 | 0.005 |
| CCI score=3+ | 0.27 | 0.06-1.10 | 0.067 |
| **Eastern Europe and Russia** |  |  |  |
| CCI score=0 | 1.00 |  |  |
| CCI score=1 | 1.16 | 0.90-1.49 | 0.259 |
| CCI score=2 | 1.39 | 1.01-1.92 | 0.042 |
| CCI score=3+ | 0.96 | 0.45-2.04 | 0.919 |
| **North America and Oceania** |  |  |  |
| CCI score=0 | 1.00 |  |  |
| CCI score=1 | 0.52 | 0.32-0.86 | 0.011 |
| CCI score=2 | 0.23 | 0.09-0.62 | 0.004 |
| CCI score=3+ | 0.61 | 0.15-2.54 | 0.494 |
| **Middle East** |  |  |  |
| CCI score=0 | 1.00 |  |  |
| CCI score=1 | 1.24 | 1.00-1.54 | 0.050 |
| CCI score=2 | 1.17 | 0.79-1.73 | 0.434 |
| CCI score=3+ | 0.79 | 0.32-1.92 | 0.597 |
| **Asia** |  |  |  |
| CCI score=0 | 1.00 |  |  |
| CCI score=1 | 0.55 | 0.38-0.81 | 0.003 |
| CCI score=2 | 0.67 | 0.37-1.19 | 0.171 |
| CCI score=3+ | 0.17 | 0.02-1.19 | 0.074 |
| **Horn of Africa** |  |  |  |
| CCI score=0 | 1.00 |  |  |
| CCI score=1 | 1.76 | 1.09-2.86 | 0.022 |
| CCI score=2 | 0.82 | 0.26-2.62 | 0.735 |
| CCI score=3+ | NED ^b^ |  |  |
| **Rest of Africa** |  |  |  |
| CCI score=0 | 1.00 |  |  |
| CCI score=1 | 1.82 | 1.19-2.77 | 0.005 |
| CCI score=2 | 1.61 | 0.69-3.76 | 0.270 |
| CCI score=3+ | 2.19 | 0.79-6.04 | 0.130 |
| **South America** |  |  |  |
| CCI score=0 | 1.00 |  |  |
| CCI score=1 | 1.07 | 0.77-1.48 | 0.695 |
| CCI score=2 | 1.03 | 0.63-1.68 | 0.898 |
| CCI score=3+ | 1.04 | 0.45-2.38 | 0.933 |

**^a^** adjusted for sex, age, macroeconomic period factors, and duration of residence.

^b^ NED=Not Enough Data to estimate relative risks.

Supplementary Table S5. Incidence rate ratios (RR) with 95% Confidence intervals (CI) for emigration among immigrants by Charlson Comorbidity Index (CCI) score, for recorded emigration only.

|  | **RR** | **95% CI** | **p-value** |
| --- | --- | --- | --- |
| **Charlson Comorbidity Index (CCI)** |  |  |  |
| 0 | 1.00 |  |  |
| 1 | 0.83 | 0.77-0.91 | < 0.001 |
| 2 | 0.72 | 0.63-0.82 | < 0.001 |
| 3+ | 0.62 | 0.47-0.82 | 0.001 |
| **Sex** |  |  |  |
| Women | 1.00 |  |  |
| Men | 1.31 | 1.30-1.32 | < 0.001 |
| **Age (years)** |  |  |  |
| 18-25 | 1.00 |  |  |
| 26-35 | 1.06 | 1.05-1.07 | < 0.001 |
| 36-45 | 0.85 | 0.84-0.86 | < 0.001 |
| 46-55 | 0.71 | 0.70-0.72 | < 0.001 |
| 56-65 | 0.67 | 0.66-0.69 | < 0.001 |
| 66-75 | 0.70 | 0.68-0.71 | < 0.001 |
| 76+ | 0.66 | 0.64-0.67 | < 0.001 |
| **Period factors** |  |  |  |
| 1992-1996 | 1.00 |  |  |
| 1997-2002 | 0.85 | 0.84-0.86 | < 0.001 |
| 2003-2007 | 1.27 | 1.25-1.28 | < 0.001 |
| 2008-2011 | 1.67 | 1.65-1.69 | < 0.001 |
| 2012-2016 | 1.28 | 1.27-1.29 | < 0.001 |
| **Duration of residence (years)** |  |  |  |
| 0-5 | 1.00 |  |  |
| 6-10 | 0.51 | 0.51-0.52 | < 0.001 |
| 11-15 | 0.33 | 0.33-0.34 | < 0.001 |
| 16-20 | 0.22 | 0.22-0.23 | < 0.001 |
| 20+ | 0.04 | 0.04-0.04 | < 0.001 |
| **Region of origin** |  |  |  |
| Finland | 1.00 |  |  |
| Other Nordic countries | 1.61 | 1.59-1.63 | < 0.001 |
| Other EU28 countries | 0.85 | 0.83-0.86 | < 0.001 |
| Eastern Europe and Russia | 0.25 | 0.24-0.25 | < 0.001 |
| North America and Oceania | 1.10 | 1.07-1.12 | < 0.001 |
| Middle East | 0.20 | 0.19-0.20 | < 0.001 |
| Asia | 0.50 | 0.50-0.51 | < 0.001 |
| Horn of Africa | 0.25 | 0.25-0.26 | < 0.001 |
| Rest of Africa | 0.37 | 0.36-0.38 | < 0.001 |
| South America | 0.45 | 0.44-0.46 | < 0.001 |

**^a^** adjusted for sex, age, macroeconomic period factors, duration of residence, and region of origin.
